# Higher Levels of Creatine Kinase MB (CK-MB) Than Total Creatine Kinase (CK): A Biochemistry Reporting Error or an Indicator of Other Pathologies?

**DOI:** 10.7759/cureus.50792

**Published:** 2023-12-19

**Authors:** Abhra Ghosh, Priyanka Datta, Manthan Dhingra

**Affiliations:** 1 Biochemistry, Dayanand Medical College and Hospital, Ludhiana, IND; 2 Biochemistry, Nil Ratan Sircar (NRS) Medical College and Hospital, Kolkata, IND; 3 Medicine and Surgery, Dayanand Medical College and Hospital, Ludhiana, IND

**Keywords:** immunoinhibition, small-bowel injury, acute myocardial injury, creatine kinase, ckmb

## Abstract

The creatine kinase (CK) enzyme and its isoenzymes hold significant diagnostic value, appearing in distinct patterns across various tissues. The most common method for creatine kinase MB (CK-MB) estimation is based on immunoinhibition. However, this method can report falsely elevated CK-MB levels in various scenarios. Persistently elevated CK-MB levels or discrepancies between measured values and the patient's clinical condition warrant further investigation, such as total CK isoenzyme electrophoresis. This report presents a case where a patient was diagnosed with acute myocardial infarction and treated according to established guidelines. However, the presence of abdominal pain, in addition to persistently elevated CK-MB after the resolution of cardiac symptoms and a higher CK-MB to total CK ratio, suggested alternative pathologies. Thorough laboratory investigations, including quantitative CK isoenzyme electrophoresis and contrast-enhanced computed tomography (CECT) of the abdomen, followed by emergency operative intervention, led to a secondary diagnosis of acute small bowel infarction.

## Introduction

The creatine kinase (CK) enzyme and its isoenzymes hold significant diagnostic value. This enzyme consists of dimeric molecules containing subunits designated as B (for brain) and M (for muscle). There are three distinct isoenzymes: CK-MM (CK3), CK-BB (CK1), and CK-MB (CK2). Additionally, two mitochondrial isoenzymes exist, mitochondrial CK (mtCK) and cytosolic CK, which are involved in the creatine phosphate shuttle [1,2]. These isoenzymes are distributed in distinct patterns across various tissues. Notably, the heart contains approximately 70% CK-MM and 25-30% CK-MB, while skeletal muscles predominantly express CK-MM (98%) with minimal levels of CK-MB (1%). CK-BB is primarily found in the brain and smooth muscle, including vascular tissues [1-3]. The most common method used for CK-MB estimation is based on immunoinhibition. However, this method can result in falsely elevated CK-MB levels. This report describes a case in which a patient was diagnosed with acute myocardial infarction and treated according to established guidelines. Nonetheless, the presence of abdominal pain, along with persistently elevated CK-MB levels after the resolution of cardiac symptoms and a higher CK-MB to total CK ratio, prompted other pathologies. Thorough laboratory investigations, including quantitative CK isoenzyme electrophoresis and contrast-enhanced computed tomography (CECT) of the abdomen, followed by emergency operative intervention, helped to make a secondary diagnosis of acute small bowel infarction.

## Case presentation

**Figure 1:**
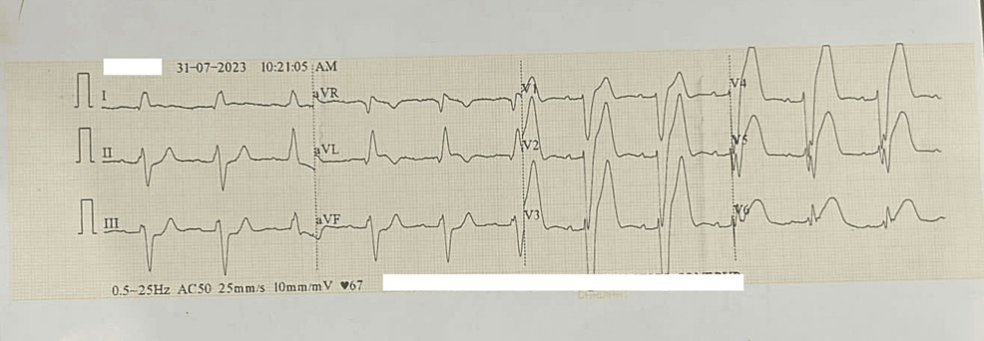
ECG of the patient showing changes suggestive of anterior wall myocardial infarction.

Recently, a 65-year-old patient presented to our ED with severe chest pain and breathlessness. At the time of admission, physical examinations revealed that the patient was hypotensive (112/68 mmHg), had a pulse rate of 68 bpm, and showed sinus arrhythmia. All peripheral pulses were palpable upon admission. The patient had a history of diabetes and hypothyroidism for the last 10 years. He was a non-smoker and non-alcoholic. His family history included hypertensive parents who were on regular medication. Based on ECG changes (Figure 1), elevated troponin T, and increased CK-MB values, a diagnosis of anterior wall myocardial infarction (AWMI) was made. Lactate levels, also measured at admission, were within normal limits (Table 1).

**Table 1:** Details of laboratory investigations at admission and during different days of stay at the hospital as the case unfolds. Trop T: Troponin T; Total CK: Total creatine kinase; CK-MB: Creatine kinase MB.

Parameters	Day 1 (on admission)	Day 2	Day 3	Day 4 (Before exploratory laparotomy)	Day 5 (Day after exploratory laparotomy)	On the day of discharge	Ref. range
Trop T, ng/mL	0.9	0.13	0.07	0.03	0.03	0.007	<0.03
Total CK, U/L	-	135	128	195	121	56	<190
CK-MB, U/L	360	256	210	275	107	20	<25
Lactate, mmol/L	0.3		-	-	1.2	-	0.3-0.8 mmol/L

The primary treatment included thrombolysis with streptokinase, followed by percutaneous coronary intervention (PCI) due to the lack of improvement in the patient's clinical condition. Subsequent examinations and clinical assessments during follow-up showed improvement in the patient's cardiac condition (Appendix 1). All vitals were stable, and the patient was doing well. Surprisingly, despite a declining trend in troponin T values over the next two days, CK-MB levels remained elevated and were relatively higher than the total CK values (Table 1).

Given the high CK-MB level compared to the total CK level, the same sample was reanalyzed using CK enzyme electrophoresis to identify any interfering substances that might have resulted in falsely elevated CK-MB results. On the third day of admission, the patient started complaining of epigastric pain, which worsened over the next 12 hours. Clinical examination revealed abdominal distension, epigastric tenderness, and absent bowel sounds. The patient was conscious, cooperative, and oriented. His vital signs included tachycardia with a pulse rate of 107 bpm, blood pressure of 124/70 mmHg, and a respiratory rate of 18/min. A CECT scan of the abdomen was performed, suggesting acute small bowel infarction (Appendix 2). Serum electrophoresis for the CK isoenzyme quantitative assay was also obtained, showing total CK at 190 U/L, CK-MM at 27%, CK-BB at 64%, and CK-MB at 9%, corroborating the CECT findings. An emergency laparotomy was performed, and a diagnosis of acute small intestine infarction was made. Resection of the gangrenous bowel with proximal jejunostomy and distal ileostomy was done. Post-operatively, Serum troponin-T, total CK, and CK-MB levels showed a downward trend. Blood lactate, measured after the laparotomy, was slightly elevated, likely due to bowel infarction, and decreased following bowel resection. The patient was monitored in the ICU post-surgery with continuous Holter monitoring and managed conservatively with IV fluids and antibiotics. Parenteral nutrition was started on postoperative day 2, along with early ambulation. Enteral feeding was resumed on postoperative day 6, followed by discharge on day 7. Blood investigation at discharge showed normal levels of total CK and CK-MB (Appendix 2, Table 1).

## Discussion

The most commonly used technique for assessing CK-MB levels is the immunoinhibition method. In this approach, the CK-M subunit is selectively inhibited using a specific antibody, and CK-B activity is measured using N-acetyl cysteine as an activator. This method has been recommended by both the German Society for Clinical Chemistry (DGKC) in 1977 and the International Federation of Clinical Chemistry (IFCC) in 2002 [4]. Specific antibodies targeting CK-M are employed to effectively inhibit approximately 99% of the catalytic activity of CK-M subunits in the sample while leaving CK-B subunits unaffected. The remaining CK-B activity, which represents about half of the CK-MB activity, is quantified using the total CK method [5]. Since the CK-BB isoenzyme is rarely detected in serum, the catalytic activities of the CK-M and CK-B subunits are closely aligned. The catalytic activity of the CK-MB isoenzyme can be calculated by doubling the measured CK-B activity [4]. For our analysis, we used CK-MB and total CK assays manufactured by Roche Diagnostics Corporation on the Roche 8000 platform, which employs the same principle of estimation. It is important to note that the widely employed immunoinhibition method may yield falsely elevated CK-MB results in various scenarios. These include a) in healthy infants and children with high CK-BB levels due to instances of central nervous system damage, tumors, childbirth, and intestinal infarction [3,6-8]; b) the presence of macro-CK types 1 and 2, observed in various conditions such as autoimmune disorders, chronic illnesses, and malignancies, including small-cell lung cancer, gastrointestinal cancer, and prostatic carcinoma [9].

Studies have suggested that elevated CK-BB levels can serve as a diagnostic marker for acute intestinal or mesenteric infarction [3,7]. In our patient, the persistently elevated CK-MB value, despite the relief of cardiac symptoms, prompted the need to investigate potential alternative causes of raised CK-MB levels. In the later phases of the disease progression, electrophoresis substantiated that the elevated CK-BB was responsible for interfering with the estimation of CK-MB levels, resulting in falsely elevated readings. Additionally, we deduced that the increase in CK-BB was caused by its release from gut smooth muscle cells that had suffered ischemic cellular damage [3,7].

We contend that in cases of discrepancy between CK-MB levels and a patient's clinical condition, total CK-MB levels must be assessed through electrophoresis or, preferably, be preceded by an immunoprecipitation step before electrophoresis. Immunoprecipitation is an additional immunological method that employs precipitating antibodies against CK-MM or CK-BB [10]. While this approach is specific and sensitive, it requires several hours to precipitate immune complexes and is therefore not recommended for emergency laboratory analyses.

## Conclusions

Doctors need to be aware of potential reasons for an increased CK-MB value when no cardiac pathologies are present, to avoid unnecessary loss of valuable time and resources. This awareness will enhance the application of the prognostic value of variant CK isoenzymes. In such cases, it is advisable to: a) determine which method was used for the estimation of CK-MB, b) take a comprehensive history from the patient to assess the likelihood of the presence of CK-MB/Macro CK in the serum, and c) confirm the presence of various isoenzymes and atypical forms of CK in the serum through electrophoresis.

## Figures and Tables

**Figure 2:**
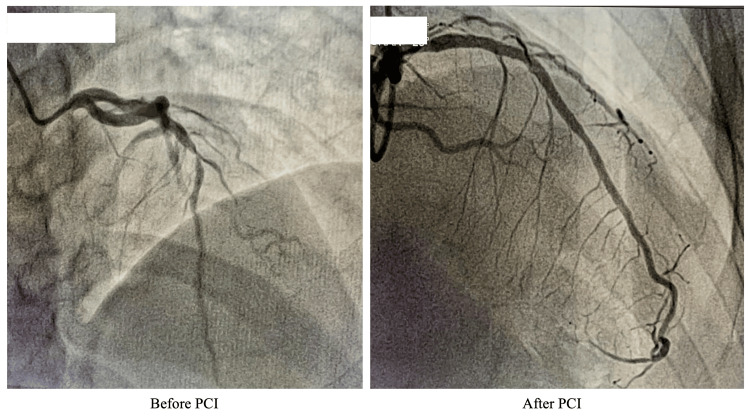
Image of pre-PCI and post-PCI angiography. All the identities of the patient and cath lab have been removed for potential conflict of interest. PCI: Percutaneous coronary intervention.

**Figure 3:**
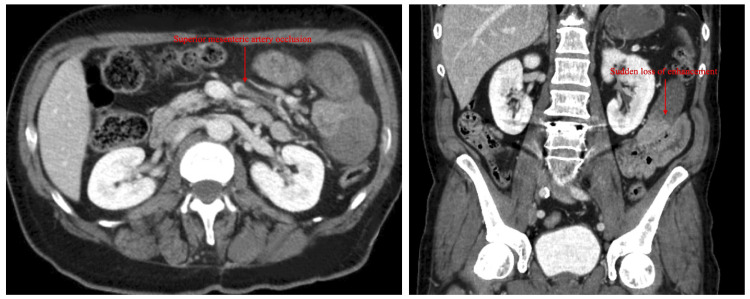
Image of CT scan of abdomen showing SMA occlusion. All the identities of the patient and the imaging centre have been removed for potential conflict of interest. SMA: Superior mesenteric artery.

## Appendices

Appendix 1

Appendix 2 
